# Ectopic expression of the *Stabilin2* gene triggered by an intracisternal A particle (IAP) element in DBA/2J strain of mice

**DOI:** 10.1007/s00335-019-09824-1

**Published:** 2020-01-07

**Authors:** Nobuyo Maeda-Smithies, Sylvia Hiller, Sharlene Dong, Hyung-Suk Kim, Brian J. Bennett, Yukako Kayashima

**Affiliations:** 1 Department of Pathology and Laboratory Medicine, University of North Carolina At Chapel Hill, CB#7525, 701 Brinkhous-Bullitt Building, Chapel Hill, NC 27599-7525 USA; 2 Department of Genetics, University of North Carolina At Chapel Hill, Chapel Hill, NC USA; 3 Present Address: Wake Forest University Medical School, Winston-Salem, USA; 4 Present Address: Obesity and Metabolism Unit, Agricultural Research Service Western Human Nutrition Research Center, US Department of Agriculture, Davis, CA USA

## Abstract

*Stabilin2* (*Stab2*) encodes a large transmembrane protein which is predominantly expressed in the liver sinusoidal endothelial cells (LSECs) and functions as a scavenger receptor for various macromolecules including hyaluronans (HA). In DBA/2J mice, plasma HA concentration is ten times higher than in 129S6 or C57BL/6J mice, and this phenotype is genetically linked to the *Stab2* locus. *Stab2* mRNA in the LSECs was significantly lower in DBA/2J than in 129S6, leading to reduced STAB2 proteins in the DBA/2J LSECs. We found a retrovirus-derived transposable element, intracisternal A particle (IAP), in the promoter region of *Stab2*^*DBA*^ which likely interferes with normal expression in the LSECs. In contrast, in other tissues of DBA/2J mice, the IAP drives high ectopic *Stab2*^*DBA*^ transcription starting within the 5′ long terminal repeat of IAP in a reverse orientation and continuing through the downstream *Stab2*^*DBA*^. Ectopic transcription requires the *Stab2*-IAP element but is dominantly suppressed by the presence of loci on 59.7–73.0 Mb of chromosome (Chr) 13 from C57BL/6J, while the same region in 129S6 requires additional loci for complete suppression. Chr13:59.9–73 Mb contains a large number of genes encoding Krüppel-associated box-domain zinc-finger proteins that target transposable elements-derived sequences and repress their expression. Despite the high amount of ectopic *Stab2*^*DBA*^ transcript in tissues other than liver, STAB2 protein was undetectable and unlikely to contribute to the plasma HA levels of DBA/2J mice. Nevertheless, the IAP insertion and its effects on the transcription of the downstream *Stab2*^*DBA*^ exemplify that stochastic evolutional events could significantly influence susceptibility to complex but common diseases.

**Electronic supplementary material:**

The online version of this article (10.1007/s00335-019-09824-1) contains supplementary material, which is available to authorized users.

## Introduction

*Stabilin 2* (*Stab2*, ID:192188) encodes a type I transmembrane receptor which is predominantly expressed in the sinusoidal endothelial cells of the liver, spleen and lymph nodes, and also at lower levels in other tissues such as kidney and heart valves (Falkowski et al. 2003; Zhou et al. 2000). Consisting of 7 fasciclin, 17 epidermal growth factor (EGF)-like, and 2 laminin-type EGF-like domains, as well as a Hyaluronan (HA)-binding X-Link domain, STAB2 functions as a scavenger receptor for various macromolecules, including HA, heparin, modified low-density lipoprotein (LDL), and pro-collagen peptides (Harris et al. 2008; Politz et al. 2002). Many other scavenger receptors including STAB1, a homologue of STAB2 that is expressed ubiquitously at lower levels, also clear many of these ligands. However, the major clearance of HA is mediated uniquely by STAB2 via the clathrin-coated pit pathway (Hansen et al. 2005). Mice lacking *Stab2* exhibit more than tenfold higher HA levels in circulation than wild-type mice, indicating the critical role of STAB2 in the systemic clearance of HA from the body (Hirose et al. 2012; Schledzewski et al. 2011).

HA is a glycosaminoglycan composed of repetitive units of disaccharide, d-glucuronic acid and *N*-acetyl-d-glucosamine. HA is abundantly present in the extracellular matrix, as well as skin, synovial fluid, vitreous body, and cartilage, where HA retains many water molecules and increases the viscosity of the fluids (Lennon and Singleton 2011; Monslow et al. 2015). Recent studies have shown that HA polymer length and quantity is also associated with many pathological processes such as inflammation and cancer metastasis (Bohaumilitzky et al. 2017; Lennon and Singleton 2011).

We previously reported that plasma HA concentration is at least ten times higher in mice on a DBA/2J background than in those on a 129S6 or C57BL/6J background. DBA/2J allele of *Stab2* locus (*Stab2*^*DBA*^) was linked to the elevation of plasma HA concentration, as well as the protection from atherosclerosis in the F2 population of DBA/2J-*Apoe*^−/−^ × 129S6-*Apoe*^−/−^ intercross (Kayashima et al. 2015). We also showed that the *Stab2*^*DBA*^ mRNA is ectopically upregulated in extrahepatic organs, such as the aorta, macrophages, heart and kidney, where little or no expression of 129 or B6 allele of *Stab2* (*Stab2*^*129*^ or *Stab2*^*B6*^*)* was detected (Kayashima et al. 2015). However, the molecular basis of ectopic expression of *Stab2*^*DBA*^ and its physiological consequences have not been explained. In this paper, we examined the genomic differences of *Stab2*^*DBA*^ allele, identified an insertion of an intracisternal A particle (IAP), a retrovirus-like element, and explored its regulatory effects on STAB2 expression.

## Materials and methods

### Mice

DBA/2J and C57BL/6J mice were purchased from the Jackson Laboratory, and 129S6/SvEvTac from Taconic Biosciences. Mice were fed regular mouse chow (Teklad global soy protein-free extruded rodent diet, irradiated, 2920X, Harlan Laboratories) and handled under protocols approved by the Institutional Animal Care and Use Committees (IACUC) of the University of North Carolina at Chapel Hill (protocol number: 17–021). Mice were anesthetized with isoflurane or avertin (2,2,2 tribromoethanol at 0.3 mg/g) to minimize discomfort, distress and pain. Carbon dioxide or an overdose of avertin were used to euthanize mice, followed by cervical dislocation.

### Cloning and sequencing of the 3′ and 5′ ends of *Stab2*-IAP

A 700 bp DNA fragment from the C57BL/6J genome that corresponds to the 500 bp upstream and 200 bp downstream of the TE insertion point in DBA/2J was made using PCR amplification. Southern blots of genomic DNA isolated from the liver of DBA/2J and 129S6 mice digested with various restriction enzymes were probed with this 700 bp fragment and indicated that the 3′ end of the TE is in the 1.2 kb EcoR1 fragment (Fig. S1). To clone the 3′ end of the insertion element, genomic DNA from DBA/2J mice was digested with EcoR1, diluted and ligated at a low concentration (5 ng/μl). The self-ligated circular DNAs were PCR-amplified using a forward primer corresponding to the sequence near the EcoR1 site in the intron 1 of *Stab2* (5′a in Fig. S2) and a reverse primer corresponding to the sequence in the promoter region of *Stab2* (5′b in Fig. S2). The 660 bp PCR product was cleaned using QIAquick PCR purification kit (Qiagen) and then directly sequenced. The 600 bp EcoR1/Bgl2 fragment from the PCR product was cloned into the pBluescript SK(+) vector (Stratagene) and its sequence was verified. The same strategy was used to clone the 5′ end of the insertion, except that Pci1 was used for digestion of genomic DNA, and primers 3′a and 3′b were used to amplify the fragment (Fig. S2). The primers used for the PCR reactions are shown in Fig. S2 and Table S1.

### Bisulfite sequencing

Genomic DNA was isolated from tissues using a conventional procedure and cleaned with phenol–chloroform extractions followed by precipitation with ethanol. Bisulfite conversion of unmethylated cytosines was done using the Epitect Bisulfite Kit from Qiagen following their protocol. The PCR reactions were set up using a left primer corresponding to the IAP sequence downstream of the 5′LTR, and the right sequence corresponded to the *Stab2* promoter region (Table S1). Reactions were carried out with 40 cycles of 1 min at 93 °C, 30 s at 58 °C and 2 min at 68 °C. The 550 bp fragments amplified were directly cloned into T vectors (Promega) or reamplified using the right and left primers containing Spe1 and BamH1 sites, respectively, and the Spe1-BamH1 fragment was inserted into Xba1 and BamH1 sites of a Bluescript vector.

### Luciferase assay

DNA fragments corresponding to − 708 to − 14 upstream from the translation initiation site of the *Stab2* gene were amplified from the 129S6 genomic DNA using promoter primer sequences 1 and 2 (Table S1), and cloned into pMCS-Cypridina Luc vector (Thermo Fisher Scientific). The EcoR1/Bgl2 fragments described above in the promoter region of *Stab2* were also amplified from the DBA/2J genomic DNA. Plasmid DNA from three independent colonies of each construct was prepared and DNA sequences were verified. HEK293T cells (ATCC) were transfected with the control empty plasmid or *Stab2*-Luc plasmids using FuGENE HD (Promega). Twenty-four hours after transfection, luciferase activities in the media were measured using Pierce™ Cypridina Luciferase Glow Assay Kit (Thermo Fisher Scientific) according to the manufacturer’s protocol. For normalization of the transfection efficiency of each well, pTK-Gaussia-Dura Luc DNA (Thermo Fisher Scientific) was co-transfected as a positive control and assayed using Pierce™ Gaussia Luciferase Glow Assay Kit (Thermo Fisher Scientific). As a negative control, luciferase plasmid without a promoter was included in each transfection experiment. The mean value of luciferase activity of the negative control plasmid (*n* = 3–5) was subtracted from the activity value of each sample. The values were then normalized to the mean activities of TK-luc plasmid, a luciferase gene driven by a promoter of the thiamine kinase gene from *Herpes simplex*. Experiments were carried out using 4–6 transfections for each construct in 96 wells and were repeated at least three times. Mean ± SE of the average activities are reported.

**Table 1:** Percent methylation of the CpG sites in the 5′-LTR sequences of *Stab2*-IAP

Mouse	# of clones sequenced	Total (25 CpG) (%)	U3 (10 CpG) (%)	R-U5-P (15 CpG) (%)	U5 only (5CpG) (%)
DBA inbred	24	75.7	62.5	84.4	80.8
Stab2DD	16	85.0	60.0	97.5	98.8

### Rapid amplification of 5′ cDNA ends (5′RACE)

5′RACE was performed as previously described (Scotto-Lavino et al. 2006). Briefly, total RNA was extracted from the liver of 129S6 and DBA/2J mice using RNeasy mini kit (Qiagen). First strand cDNA was synthesized by SuperScript III (Life Technologies) using gene specific primer 1 (GSP1), which is specific to the sequences in exon 2 of *Stab2*. After the template RNA was digested with RNaseH (Life Technologies), cDNA was tailed with poly dCTP by TdT, then PCR-amplified using gene specific primer 2 (GSP2), which is specific to the exon 1 of *Stab2* and contains a Xho1 site, and an anchor primer that anneals to the poly dCTP tail and contains a Mlu 1 site. The PCR products were directly sequenced, or sequenced after cloning into the Mlu1–Xho1 site of pCMV6-Entry vector (Origene). The primers used were shown in Table S1.

### Isolation of LSECs from the liver

LSECs of the liver and hepatocytes were separated as shown in Fig. S3, following the protocol previously described (Bartneck et al. 2015; Meyer et al. 2016). Briefly, two to three month old male mice were anesthetized with isoflurane, a catheter was inserted from the right atrium into the supra-hepatic portion of the inferior *vena cava*, and the liver was perfused with pre-warmed wash buffer (calcium-free Hank’s Balanced Salt Solution (HBSS) supplemented with 5 UI/ml heparin, 0.1% glucose, 25 mM HEPES, 0.5 mM EGTA, 100 UI/ml penicillin and 100 μg/ml streptomycin) for 5 min at a flow rate of 5 ml/minute, then with digestion buffer (Iscove’s modified eagle medium (IMDM) with Glutamax containing 80 U/ml type IV collagenase (Thermo Fisher Scientific) and 0.08 μg/ml DNase I (Sigma)) for 5 min. The liver was removed and minced in post-digestion solution (Dulbecco’s modified eagle’s medium (DMEM) with 5% fetal bovine serum (FBS), 100 UI/ml penicillin and 100 μg/ml streptomycin). The liver suspension was filtered through a 70 μm strainer and centrifuged at 68×*g* for 5 min to remove most of the hepatocytes. The supernatant was centrifuged at 600×*g* for 10 min and the pellet was resuspended in 17.6% Optiprep (Sigma). The cell suspension was layered with 8.2% Optiprep and centrifuged at 1400×*g* for 30 min. The interphase layer enriched with LSECs and macrophages was collected, suspended into magnetic-activated cell sorting (MACS) buffer (calcium-free Dulbecco’s phosphate buffered saline (DPBS) with 0.5% FBS and 2 mM EDTA), and centrifuged at 780×*g* for 10 min. The cell pellet was resuspended in MACS buffer, and LSECs were further purified by the CD11b-positive cell depletion using anti-CD11b microbeads (Miltenyl Biotec). After the LSECs were plated and incubated at 37 °C for 2 h in the mouse endothelial cell medium (Cell Biologics), non-adherent cells were removed by washing with PBS. Attached cells were used for further analyses.

### Flow cytometry

Cells were blocked with Fc Blocker (anti-mouse CD16/32, BioLegend) and stained with Alexa Fluor 488-labeled rat monoclonal anti-mouse Stabilin 2 (1:100, clone #34-2, MBL) and Allophycocyanin (APC)-labeled rat anti-mouse F4/80 (1:500, clone BM8, eBioscience) antibodies. Dead cells were discriminated by DAPI staining. Fluorescence was measured on Attune NxT (Thermo Fisher Scientific) and assessed by FlowJo software.

### Quantitative RT-PCR

Total RNA was isolated using RNeasy mini kit (Qiagen) according to the manufacturer’s instructions. Quantitative RT-PCR was performed by the 7500 Real Time PCR system (Applied Biosystems) with one-step protocol containing MultiScribe reverse transcriptase (Thermo Fisher Scientific) for RNA quantification. The primers for the detection of *Stab2* transcripts were designed to span across the splice junction between exon 50 and 51. The sequences of the primers and the probes were shown in Table S1.

### Western blot

Mice were intracardially perfused with DPBS and tissues were isolated and snap frozen in liquid nitrogen. Tissue samples were homogenized in radioimmunoprecipitation assay (RIPA) buffer (50 mM Tris, pH 8.0, 150 mM NaCl, 1% v/v Triton X-100, 0.5% v/v sodium deoxycholate, 0.1% SDS) supplemented with cOmplete Mini protease inhibitor cocktail (Roche). Cells were lysed in RIPA buffer. Samples were separated on 10% SDS-polyacrylamide gels (Bio-Rad) and transferred to PVDF membranes (Millipore). After blocking in 2% Amersham ECL blocking regent (GE Healthcare Life Sciences), membranes were incubated with goat polyclonal anti-mouse Stabilin 2 (1:500, clone M20, Santa Cruz), followed by incubation with peroxidase-conjugated donkey anti-goat antibody IgG (1:5000, Santa Cruz), then developed with SuperSignal West Pico Chemiluminescent Substrate (Thermo Fisher scientific).

### Immunostaining

For immunostaining of the cells, samples were fixed with 4% PFA, permeabilized with 0.1% Triton-X, blocked with 5% goat serum in PBS, and incubated at 4 °C for overnight with goat polyclonal anti-mouse Stabilin 2 (1:50, clone M20, Santa Cruz) and rat monoclonal anti-mouse F4/80 (1:100, clone C1:A3-1, Bio-Rad) antibodies or mouse monoclonal anti-DDK antibody (1:1000, clone 4C5, OriGene). The cells were washed with PBS and then incubated with Alexa Fluor 488-conjugated donkey anti-goat and Alexa Fluor 594-conjugated donkey anti-rat antibodies, or Alexa Fluor 594-conjugated goat anti-mouse antibody (1:500, Thermo Fisher Scientific).

For immunostaining of the tissues, samples were embedded in O.C.T. compound (Tissue-Tek) and frozen in liquid nitrogen. Frozen sections (7 μm) were air dried, fixed in cold acetone, and blocked with 5% normal goat serum. Slides were incubated with primary antibodies: rabbit polyclonal anti-STAB2 (1:500, a kind gift from Dr. Sergij Goerdt at the University of Heidelberg) (Falkowski et al. 2003) and rat monoclonal anti-mouse LYVE1 (1:100, clone ALY7, Novus Biologicals), followed by incubation with secondary antibodies: Alexa Fluor 594-conjugated goat anti-rabbit IgG (1:500, Thermo Fisher Scientific) and Alexa Fluor 488-conjugated goat anti-rat IgG (1:500, Thermo Fisher Scientific). Images were captured with IX81 fluorescence microscope (Olympus) or LSM710 laser scanning confocal microscope (ZEISS).

### Statistics

Values were indicated as mean ± SE. Comparisons between samples were done by one-way analysis of variance (ANOVA) followed by Tukey–Kramer’s HSD test. Data were analyzed using JMP software version 9.0 (SAS Institute) and SigmaPlot 11.2 software (Systat).

## Results

### Insertion of an IAP element within the promoter region of the *Stab2*^*DBA*^ gene

The unique expression pattern of *Stab2* in DBA/2J prompted us to compare the genomic sequences of the *Stab2* gene between DBA/2J and other inbred strains using publicly available data from the Sanger Institute Mouse Genome Project (Keane et al. 2011; Yalcin et al. 2011). The comparison revealed that an insertion of a transposable element (TE) is present at 220 bp upstream of the translation start site in the *Stab2*^*DBA*^ allele, but not in the *Stab2*^*129*^, *Stab2*^*B6*^ allele or in other commonly used inbred strains (Fig. S1) (Keane et al. 2011; Quinlan et al. 2010).

**Fig. 1:**
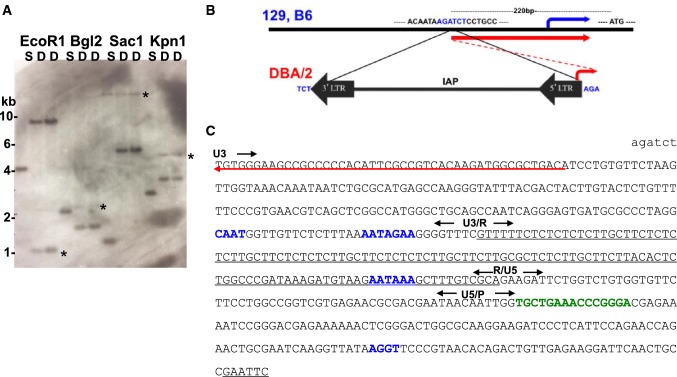
The intracisternal A particle (IAP) element inserted in the promoter region of *Stab2*^*DBA*^. **a** An example of Southern blots of the genomic DNA from 129S6 (S) and DBA/2J (D). The DNA probe is 700 bp, 500 bp of which corresponds to 5′ to the insertion site (Bgl2) while 200 bp corresponds to 3′ to the insertion site. Consequently, intensities of fragments hybridized to the 3′ portion of the probe only (marked with *) are weaker than those hybridized to the 5′ portion. **b** Insertion of the IAP in the promoter region of the *Stab2* gene in the DBA/2J genome compared to those in the 129S6 (129) and C57BL/6J (B6). The insertion site is 220 bp upstream from the translation initiation site (ATG) and flanked by AGATCT (Bgl2 site). The blue arrow indicates the starting position of normal transcript, and the red arrows indicate the alternative transcript starting within the 5′LTR. **c** Nucleotide sequence of Bgl2/EcoR1 fragment containing 5′LTR of the *Stab2*-IAP. The transcript starting from LTR in reverse orientation is underlined with a red arrow. Starts and ends of U3, R and U5 domains are marked. P stands for post 5′LTR sequence. CAAT, ATA, AATAAA, AGGT motifs are in blue, and primer binding site complementary to the 3′ end of the Phe-tRNA is in green

Southern blot analyses of genomic DNA from DBA/2J and 129S6 with probes flanking the insertion site of the *Stab2*^*129*^ allele suggested that the TE insert in DBA/2J genome is about 5.6 kb in length, and that the 3′ end is within a 1.2 kb EcoR1 fragment (Figs. 1a, S1). We therefore cloned a 600 bp fragment containing the 3′ end of the insert from the genomic DNA of DBA/2J using a strategy to clone an insertional repeat (Fig. S2). Genomic DNA was digested with EcoR1, and the purified DNA was re-ligated and PCR-amplified using a reverse primer corresponding to the sequence in the promoter region of *Stab2* and a forward primer corresponding to the sequence near the EcoR1 site in the intron 1 of *Stab2* (Table S1). The nucleotide sequence of the fragment revealed a 5′ long terminal repeat (5′LTR) of an IAP element, which is inserted in a reverse orientation relative to the *Stab2* gene (Fig. 1b). Similar to other retroviral LTR sequences, it contains characteristic motifs including CAT-box and TATA-box in the U3 domain that initiate transcription of the viral genome in its forward direction and a poly A addition sequence in the R domain that terminate the transcripts initiated from the upstream genome (Christy and Huang 1988). The 5′LTR is followed by a primer binding domain sequence complementary to the 3′ end of Phe-tRNA and a consensus splice-donor sequence (Fig. 1c) (Ono and Ohishi 1983).

The IAP family contains approximately 1000 members in C57BL/6 mice with identical 5′ and 3′ LTRs, indicating very recent insertion events (Qin et al. 2010). While the insertionally polymorphic *Stab2*-IAP is likely a recent integration, sequencing of its 3′LTR isolated with a similar strategy revealed two nucleotide differences from the sequence of 5′LTR at the end of the U5 region (Fig. S2B). As expected for a typical IAP retrotransposition event, the *Stab2*-IAP element is flanked by AGATCT sites, a finding consistent with target site duplication (Qin et al. 2010). Interestingly, the 5′LTR sequence is completely identical to that of an IAP element in intron 6 of the *Cdk5rap1* gene on Chr2, which is present in the genomes of C57BL/6 and C3H/HeJ but not in DBA/2J nor in 129S6 genome (Druker et al. 2004). Sequence identity includes the R-domain of the LTR where a stretch of pyrimidine-rich sequence is highly variable among different IAP elements (Christy et al. 1985). Additionally, the lengths of restriction fragments containing the 3′ and 5′ ends of *Stab2*-IAP determined by the Southern blots matched well with the restriction map of the *Cdk5rap1-*IAP (Fig. S1), confirming that *Stab2*-IAP element also belongs to the IAP1Δ1 subclass. This subclass contains a 1.9 kb deletion affecting *gag* and *pol*, creating a gag-pol fusion gene and protein which facilitates its own retrotransposition (Saito et al. 2008). Notably, nearly all recent activity of the IAP family, as judged by germ line mutations due to IAP insertions, involve 1Δ1 elements (Gagnier et al. 2019). The degree of similarity between the identities of the current *Cdk5rap1-*IAP and *Stab2*-IAP suggests that a burst of IAP insertions took place in parental genome and the two IAPs were fixed differentially during the establishment of individual inbred strains in early 1900s, rather than that the transpositions occurred and fixed independently in C57BL/6J line and in DBA line (Beck et al. 2000).

### 5′LTR of the *Stab2*-IAP drives the *Stab2*^*DBA*^ gene expression in a reverse orientation

**Fig. 2:**
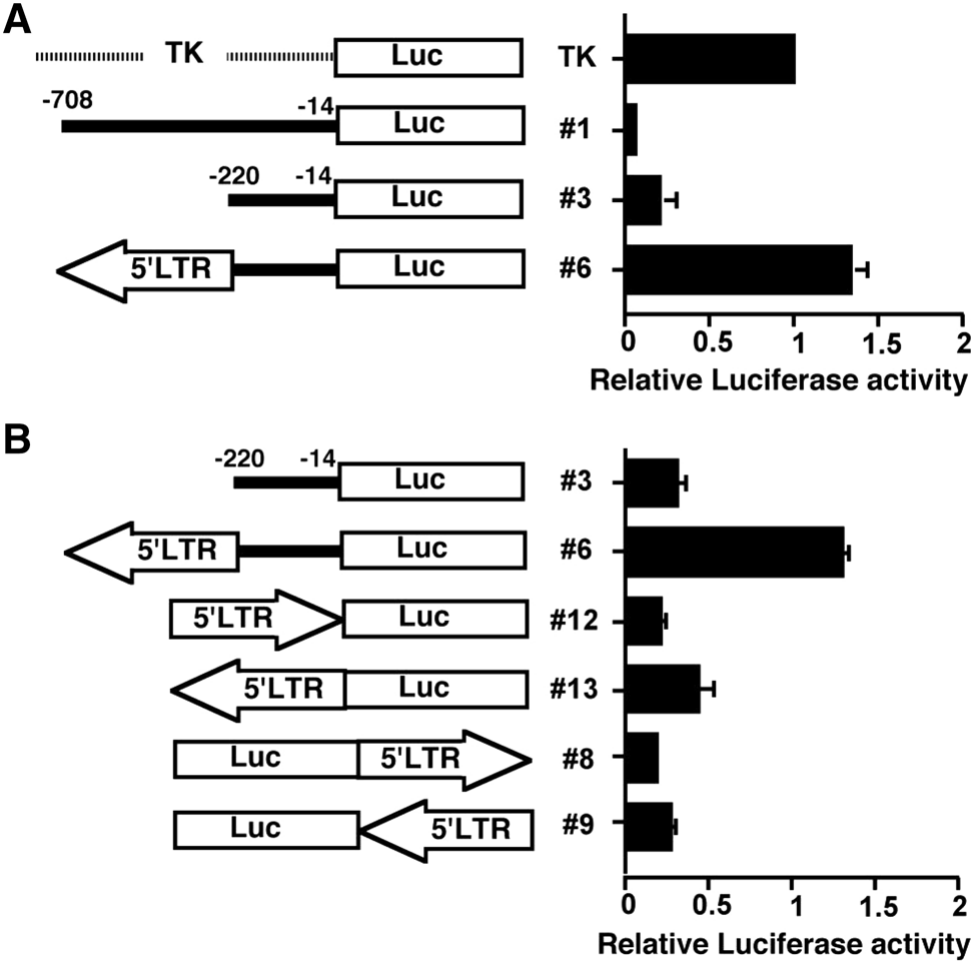
Cypridina luciferase reporter (Luc) assay in HEK293T cells. Cells were transfected with the control TK-luc plasmid or *Stab2* promoter driven Luc reporter constructs with or without the 5′LTR sequence (arrows). Activities of luciferase in the medium were measured at 24 h after transfection. Signal intensities are normalized by a Gaussia luciferase. Data are shown relative to TK-Luc intensity as 1.0. **a** Luciferase activity of a 700 bp fragment upstream of the *Stab2* gene in 129S6 and C57BL/6J (#1), of a 220 bp fragment as in the *Stab2* gene of DBA/2J (#3), and with a 5′LTR in the reverse orientation (#6). Experiments were carried out with at least 4 wells per construct in each experiment and the mean ± SE of average activities from four experiments are presented. **b** Promoter activity assessed with the 5′LTR sequence of the *Stab*-IAP inserted upstream of the promoterless Luc in both orientations (#12 and #13), and general promoter activity assessed by insertion 3′ to the promoterless Luc in both orientations (#8 and #9). Data are the mean ± SE of average activities from three experiments

High levels of *Stab*2 are normally limited to sinusoidal endothelial cells of the liver, spleen and lymph nodes (Adachi and Tsujimoto 2002; Falkowski et al. 2003). Yet, our previous work showed that the expression of *Stab2 *in other tissues such as in the heart and kidneys were 50–100 times higher in DBA/2J mice than in 129S6 and C57BL/6J mice (Kayashima et al. 2015). To examine the *Stab2* gene promoter function which would drive this ectopic expression, luciferase reporter assays in HEK293 cells were conducted. Since human genome does not contain *Stab2*-IAP like sequence, it is unlikely that the genome of HEK293 cells has acquired mechanisms that specifically repress the *Stab2*-IAP functions in transient expression experiments. In the first set of experiments, we compared the DNA fragments upstream of the *Stab2*^*129*^ and *Stab2*^*DBA*^ alleles and found that the DNA fragment corresponding to -220 to -14 bp upstream from the translation initiation site contains a basal promoter of the *Stab2* gene with small but detectable luciferase activity (#3 in Fig. 2a). On the other hand, the reporter plasmid with -708 to -14 bp fragment of the *Stab2*^*129*^ allele showed dramatically low luciferase activity (#1 in Fig. 2a). This result suggests that a transcriptional repressor must be present immediately upstream to the basal promoter, accounting in part for the silencing of the *Stab2* gene in general, such as in HEK293 cells. In contrast, the addition of a 500 bp EcoR1/Bgl2 fragment containing the 5′LTR to the 220 bp of minimum promoter sequence in a reverse orientation, as in the *Stab2*^*DBA*^ allele, caused an increase in luciferase activity of fivefold over that of the 220 bp minimum *Stab2* promoter (#6 in Fig. 2a). Retroviral LTRs contain multiple motifs necessary for transcriptional regulation as described above. However, in our second set of experiments (Fig. 2b), promoter activity of the 5′LTR alone with no basal promoter is approximately 30% in the reverse orientation relative to the reporter gene (#13), and 15% in the same orientation (#12), compared to the genomic context (#6, with the 220 bp minimum *Stab2* gene promoter), indicating that the 5′LTR by itself is not sufficient for robust promoter function. Similarly, luciferase activity was not appreciably increased when the 5′LTR was incorporated at the 3′ end of the luciferase gene driven by the minimum promoter in either orientation (#8, #9 in Fig. 2b) or by introducing it upstream of the thiamine kinase promoter driven reporter (TK-luc) plasmid (not shown). Thus, our results suggest that the LTR sequence is not acting as a general enhancer.

**Fig. 3:**
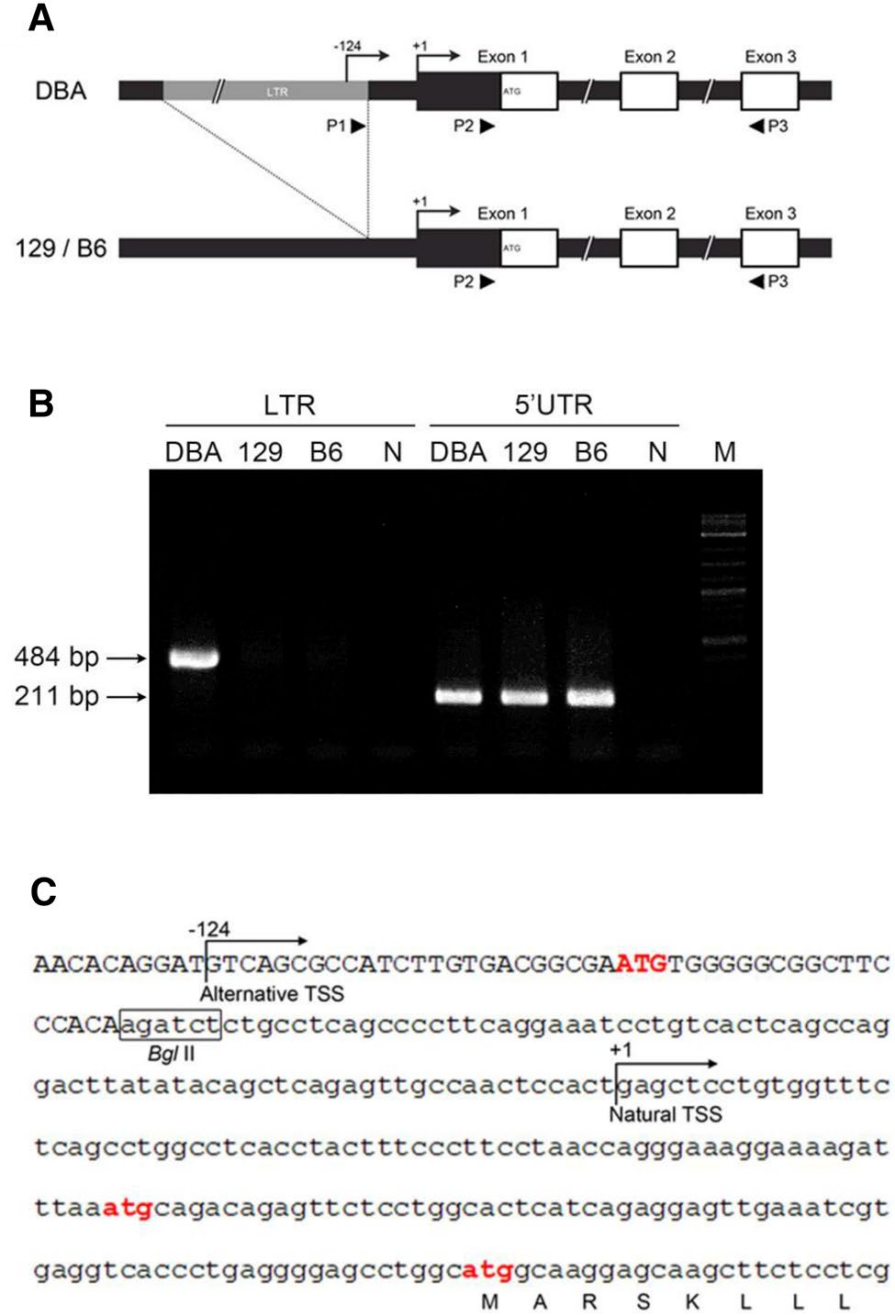
IAP-driven transcripts of *Stab2*^*DBA*^. **a** Location of primers for RT-PCR used to detect the IAP-driven transcripts of *Stab2* (arrowheads P1 and P3) and those that detect both the normal and the IAP-driven transcripts (P2 and P3) are shown. **b** RT-PCR of RNA from the liver of DBA/2J (DBA), 129S6 (129) and C57BL/6J (B6) mice using primers shown in **a**. *N* negative control without RNA, *M* DNA size markers. **c** Sequence of the 5′ upstream region of the *Stab2*^*DBA*^ gene. 5′LTR of the IAP is shown in upper case; Bgl2 site is boxed; ATG codons are highlighted in red; position of the natural (+ 1) and alternative (− 124) transcription start sites (TSSs) determined by 5′RACE analyses are indicated

Although the IAP is in an opposite transcriptional orientation relative to that of *Stab2*^*DBA*^, an antisense promoter within the 5′LTR can initiate reverse-oriented transcription and cause disturbance of the normal transcription of nearby genes (Chuong et al. 2017; Gagnier et al. 2019; Thompson et al. 2016). To confirm that the *Stab2*^*DBA*^ transcription is driven by the 5′LTR in vivo, we performed reverse transcription PCR using RNA from tissues using a forward primer corresponding to a sequence within the 5′LTR and a reverse primer corresponding to a sequence in the *Stab2* exon 3 (Fig. 3a). A single fragment of expected size (484 bp) for transcripts initiated within the 5′LTR was detected in the liver of DBA/2J mice but not in that of 129S6 and C57BL/6J (Fig. 3b). In contrast, 211 bp products expected from both the LTR-driven and normal transcripts were detected by a forward primer corresponding to the 5′ untranslated region of the *Stab2* mRNA in the tissues of DBA/2J, 129S6 and C57BL/6J mice (Fig. 3b). Rapid amplification of 5′ cDNA ends (5′-RACE) using liver mRNA revealed that there is an alternative transcription start site (TSS) within the 5′LTR in DBA/2J, in addition to the natural TSS (Figs. 2a, 3c). The alternative TSS is located at − 124 bp upstream of the normal TSS (Fig. 3c) of the *Stab2* gene. These experiments, although not quantitative, clearly demonstrate that the 5′LTR of the IAP element in reverse orientation drives the *Stab2*^*DBA*^ gene transcription ectopically in vivo*.*

### A locus on the chromosome 13 of C57BL/6J suppresses ectopic *Stab2*^*DBA*^ expression

**Fig. 4:**
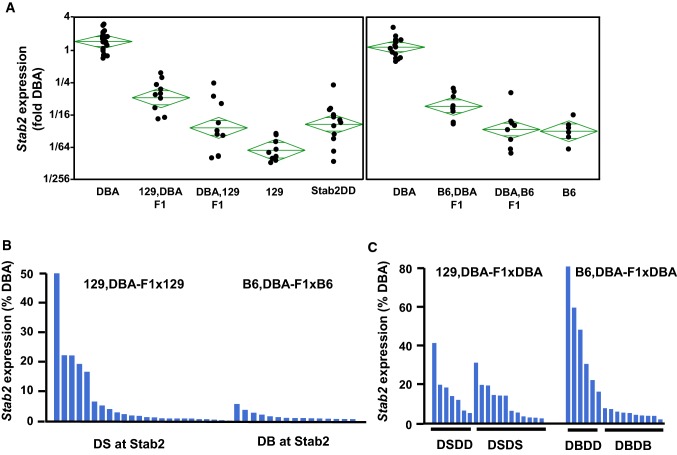
Effects of genetic backgrounds on the ectopic *Stab2* gene expression in the hearts. Hearts of 3 weeks old mice were isolated and the *Stab2* mRNA levels were determined by RT-PCR. **a** Expression relative to the hearts of DBA/2J mice was expressed as fold difference in log2 scale to emphasize the genetic background effects. Left panel: F1 mice were generated from crosses of 129S6 females and DBA/2J males (129,DBA-F1) or DBA/2J females and 129S6 males (DBA,129-F1). Note F1 mice all carry one copy of DBA/2J genome and one copy of 129S6 genome. Stab2DD is a congenic strain in which the *Stab2*^*DBA*^ allele was transferred onto a 129S6 genetic background by more than 6 generations of serial backcrosses and are homozygous for *Stab2*^*DBA*^. Right panel: F1 mice were generated from crosses of C57BL/6J females and DBA/2J males (B6,DBA-F1) or DBA/2J females and C57BL/6J males (DBA,B6-F1). Green diamonds illustrates a group mean (middle horizontal bars) and confidence intervals (height of the diamonds). **b** Relative expression levels (%DBA) in the hearts of individual mice heterozygous for the *Stab2*^*DBA*^ allele derived from 129,DBA-F1 females backcrossed to 129S6 males (DS at *Stab2*) and from B6,DBA-F1 females backcrossed to C57BL/6J males (DB at *Stab2*). All mice presented have at least one copy of 129S6 (or C57BL/6J) at other parts of genome. **c** Expression levels (%DBA) in the hearts of individual mice heterozygous for *Stab2*^*DBA*^ allele derived from 129,DBA-F1 females or B6,DBA-F1 females backcrossed to DBA/2J males. Only those heterozygous for *Stab2*^*DBA*^ are presented. All mice have at least one copy of DBA genome but randomly carry segments of DBA/2J genome. Genotypes of *Stab2* at Chr10 and a Chr13:67 Mb marker were determined. DSDD and DBDD indicate heterozygotes for *Stab2*^*DBA*^ and homozygous for Chr13:66 Mb of DBA/2J. DSDS and DBDB indicate heterozygotes at both loci

We placed the *Stab2*^*DBA*^ allele onto a 129S6 background through a series of backcrossing and established a congenic line homozygous for the *Stab2*^*DBA*^ allele, which we designate as Stab2DD. Surprisingly, *Stab2* transcripts were practically undetectable in the hearts or kidneys of Stab2DD mice (Fig. 4a, left panel). This observation suggests that a locus (or loci) other than those cis to the *Stab2* gene on Chr10 is regulating the ectopic expression of the *Stab2*^*DBA*^ gene. To further explore the nature of this control, we crossed DBA/2J females with 129S6 males or vice versa, and DBA/2J females with C57BL/6J males or vice versa, and *Stab2* gene expression in the hearts of their offspring were measured by quantitative RT-PCR. The expression levels in the F1 heterozygotes were less than half of the levels expected in obligatory heterozygotes of the *Stab2* gene in crosses between 129S6 females and DBA/2J males (129,DBA-F1), while less than one eighth in crosses between DBA/2J females and 129S6 males (DBA,129-F1) (Fig. 4a, left panel). Similarly, in the hearts of F1 mice between DBA/2J and C57BL/6J, the *Stab2* expression was much reduced compared to DBA/2J mice and lower when DBA genome was from females (164th of expected levels) than when it was from males (one sixteenth) (Fig. 4a, right panel). Collectively, these data indicate that the ectopic expression of *Stab2* is differentially controlled by other loci depending on the strain- and male/female differences.

**Fig. 5:**
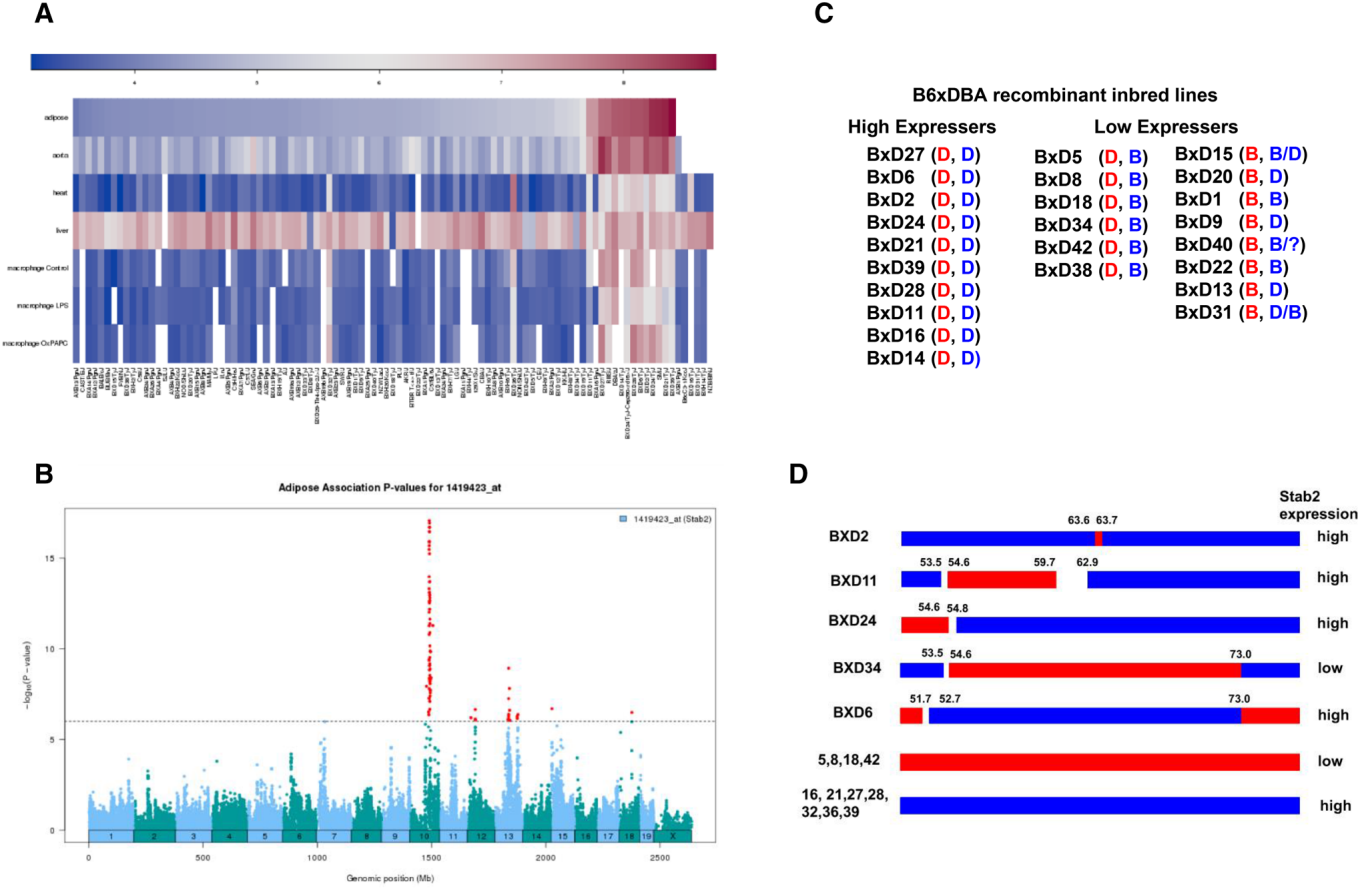
Suppression of ectopic expression of *Stab2*^*DBA*^ by Chr13:59.7–73.0 Mb of C57BL/6J. **a** Expression heatmap of the *Stab2* gene in various tissues of inbred mice represented in the Hybrid Diversity Panel. Taken from https://systems.genetics.ucla.edu. Expression in adipose tissue aligned from low (blue) to high (magenta) of individual lanes reveals a cluster of high expressers. **b** eQTL analysis of the Hybrid Diversity Panel also taken from https://systems.genetics.ucla.edu illustrates the association of SNPs with adipose tissue expression levels of *Stab2*. *Y* axis is – log 10 (*p* value). Significantly associated SNPs on chromosome 10 (cis) and on others (trans) are shown by red dots. **c** C57BL/6J x DBA/2J recombinant inbred lines categorized by high expressers and low expressers and their genotypes at *Stab2* (Chr10:87 Mb, rs30240632, red) and Chr13:65 Mb marker (rs3657887, blue) listed in GeneNetwork (www.genenetwork.org) are presented. **d** Recombinant map of the 50–75 Mb of chromosome 13 of the BxD recombinant inbred strains whose *Stab2* gene expression is available

Before embarking on a global breeding strategy to identify the location of factors not residing on Chr10 that affect *Stab2* expression we sought to narrow the possible genomic locations. One approach is to use gene expression from the Hybrid Diversity Panel (Lusis et al. 2016), which contains C57BL/6J, DBA/2J and recombinant inbred lines from crosses of these two strains (BxD). *Stab2* gene expression in the tissues of mice in the Hybrid Diversity Panel is publicly available from the Systems Genetics Resource database of UCLA (https://systems.genetics.ucla.edu), and a heatmap of *Stab2* in a variety of tissues including the adipose tissues, aorta, heart and macrophage showed a striking cluster of high expressers. The cluster includes three inbred strains, DBA/2J, SM/J and RIIIS/J, and some of the recombinant inbred strains derived from C57BL/6J and DBA/2J crosses (BxD) (Fig. 5a) [note that SM/J and DBA2 share common ancestry, while origin of RIIIS/J is unknown (Beck et al. 2000)]. In contrast, no specific clustering was observed in the expression in the liver. eQTL search for the adipose tissue expression of *Stab2* revealed four genome wide significant eQTL peaks in trans in addition to the strong cis eQTL associated with the *Stab2* gene (Fig. 5b). The most prominent trans eQTL peak was on Chr13. BxD genotype and chromosomal recombinant panels of the individual BxD lines were then compared using single nucleotide polymorphism (SNP) data sets available through the Center for Genome Dynamics at the Jackson Laboratories (Yang et al. 2011) which revealed that homozygosity of DBA-derived SNPs near 60–70 Mb of Chr13 is consistent with high ectopic *Stab2*^*DBA*^ gene expression. Eleven high expressers of BxD strains were all homozygous for the *Stab2*^*DBA*^ allele on Chr10 as well as homozygous for Chr13:60–70 Mb from DBA/2J (Fig. 5c). In contrast, among the low expressers, all six lines that are homozygous for the *Stab2*^*DBA*^ alleles carry Chr13:60–70 Mb of C57BL/6J, while none of the mice carrying Chr13:60–70 Mb of DBA/2J had the *Stab2*^*DBA*^ allele. The recombination maps of BxD lines of four *Stab2*^*DBA*^ high expressers and one low expresser contained informative recombinations near the region and strongly indicates that a modifier of *Stab2*^*DBA*^ is located in between the 59.7 to 73 Mb of the Chr13 of C57BL/6J (Fig. 5d).

To confirm that the B6 genome contains a repressor of *Stab2* expression, *Stab2* expression was examined in hearts of the offspring of a cross between B6,DBA-F1 females with C57BL/6J males. Remarkably, none of the 19 B6-backcrossed mice that carry one *Stab2*^*DBA*^ allele expressed the gene above the background (Fig. 4b). *Stab2*^*DBA*^ heterozygous offspring from the B6,DBA-F1 females crossed with DBA/2J males show that two copies of Chr13:59.7–73.0 Mb from DBA2 (genotyped using markers at 64, and 67 Mb) are necessary for the significant expression of *Stab2*^*DBA*^ in hearts (Fig. 4c). These results confirm that the factor(s) on Chr13:59.7–73 Mb from C57BL/6J dominantly represses the expression of *Stab2*^*DBA*^.

To address a question whether the Chr13:59.7–73.0 Mb in 129S6 modifies Stab2 expression similarly as in C57BL/6J, 129,DBA-F1 females were crossed with 129S6 males. Surprisingly, we detected significant levels of *Stab2* expression in 5 of the 25 offspring carrying a *Stab2*^*DBA*^ allele despite that they all carry at least one copy of 129 genome (Fig. 4b). Furthermore, among the *Stab2*^*DBA*^ heterozygous offspring of the 129,DBA-F1 females crossed with DBA/2J males, *Stab2* expression in half of those carrying one copy of the Chr13:59.7–73.0 Mb from DBA/2J were at similar levels as those carrying two copies from DBA/2J (Fig. 4c). Our results are compatible with a model that the repressor(s) on Chr13 of 129S6 is not sufficient and other chromosomal loci on the 129S6 genome are necessary to repress the *Stab2*^*DBA*^ gene expression completely.

### CpG methylation in 5′LTR and the *Stab2*^*DBA*^ gene expression

**Fig. 6:**
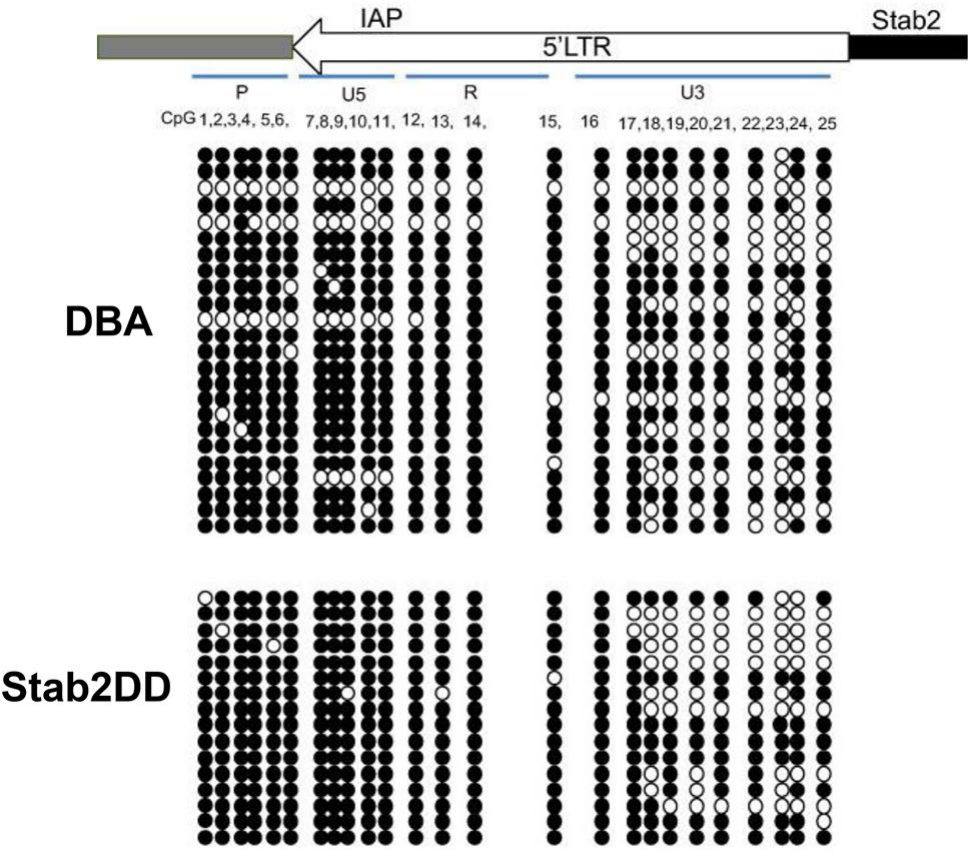
CpG methylation status of the 5′LTR of the *Stab2*-IAP in the heart DNA. Bisulfite sequencing was carried out with DNA isolated from hearts of a DBA and a backcrossed line, Stab2DD mice. A total of 25 CpG sites throughout the 5′LTR were evaluated for unmethylation (open circles) or methylation (closed circles) in each clone. The positions of CpG relative to the LTR domains are illustrated on the top

We next examined the mechanism underlying the control of *Stab2* transcription by the modifier loci. Previous reports have shown that the methylation of CpG dinucleotide within the 5′-LTR sequences of endogenous retroviral sequences (ERV) is correlated with the transcriptional repression of nearby genes (Maksakova et al. 2006; Rakyan et al. 2002). There are 25 CpGs in the 5′LTR region of the *Stab2*-IAP. Bisulfite sequencing of the genomic DNA isolated from the hearts of DBA/2J and Stab2DD mice were 75.7% and 85% respectively. Regionally, the average methylation of the 10 CpGs in U3 domain (the nearest to the start site of the *Stab2* gene) was 62% and 60%, while the average methylation of the 15 CpGs in the remaining 3′ region (R-U5-P domains) was 85% and 97% in DBA/2J and Stab2DD DNA, respectively. (Fig. 6 and Table 1). Percent methylation at each CpG site in the 3′ region differed significantly (*p* < 10^−9^). Moreover, non-methylated CpGs in the U5 domain were generally clustered, and four of 24 clones had blocks of non-methylated CpGs in DBA/2 compared to none of 16 clones from Stab2DD. Since these data show that the CpG methylation overall and in the U5 region associates with the reduced transcription of *Stab2*, the U5 domain is likely involved, at least in part, in the transcription activity of the 5′LTR.

### STAB2 protein from the LTR-driven transcript is not detectable

**Fig. 7:**
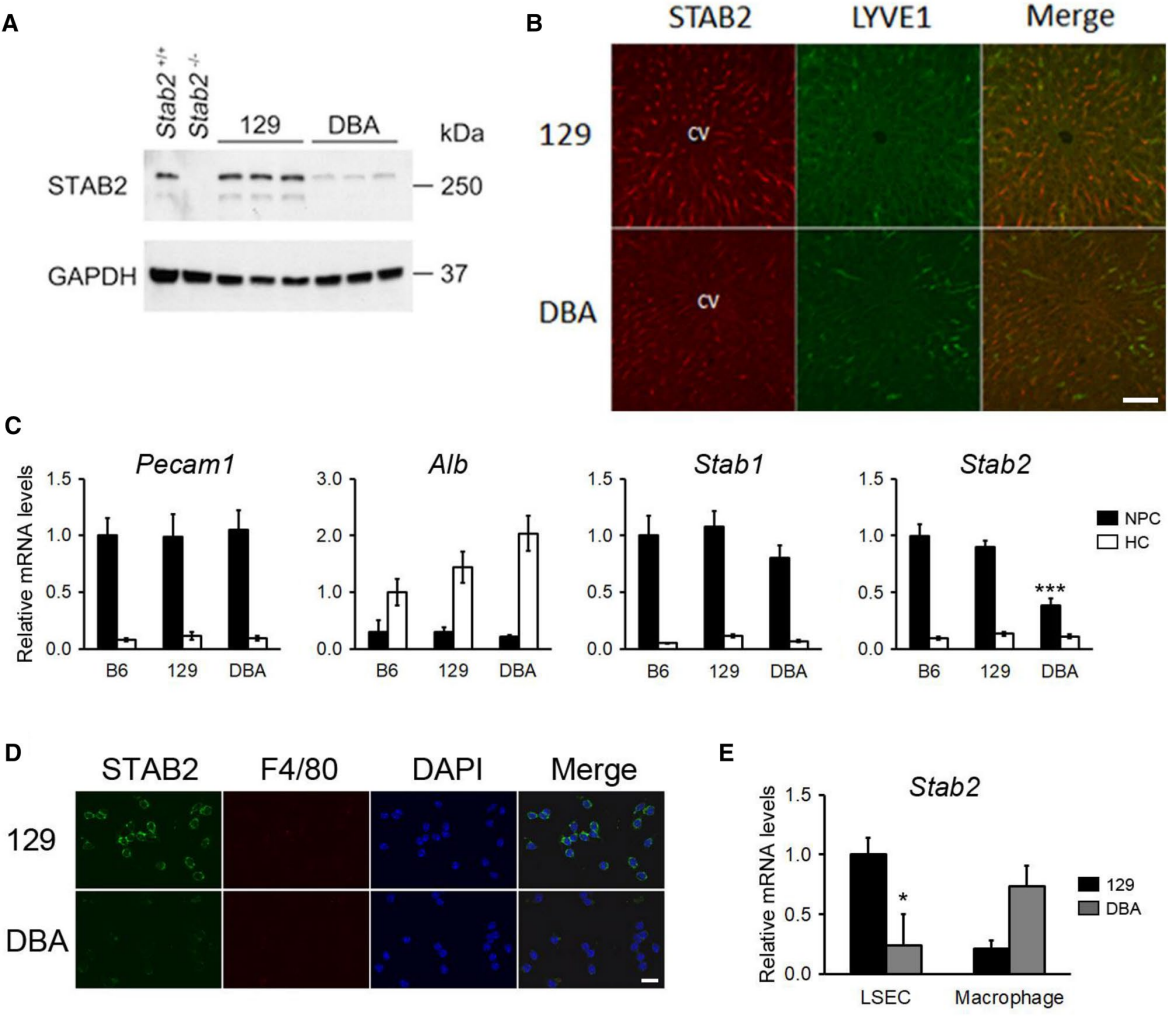
Reduced expression of *Stab2*^*DB*A^ in the liver sinusoidal endothelial cells (LSECs) of DBA/2J. **a** A western blot for STAB2 from the whole liver of 129S6 and DBA/2J mice. Liver lysates from *Stab2*^+/+^ and *Stab2*^−/−^ mice on a C57BL/6J background were used as a positive and negative control. **b** Immunohistochemical staining of STAB2 in the liver from 129S6 and DBA/2J mice. STAB2 signal was detected along sinusoids and partly overlapped with Lymphatic vessel endothelial hyaluronan receptor 1 (LYVE1). Bar = 100 μm. **c** Quantitative RT-PCR of *Pecam1*, *Albumin* (*Alb*), *Stab1* and *Stab2* in the non-parenchymal cells (NPCs) and hepatocytes (HCs) isolated form the liver of 3 months old C57BL/6J, 129S6 and DBA/2J male mice (*n* = 5 each. ***p < 0.001 vs. C57BL/6J and vs. 129S6). **d** Immunofluorescence staining for STAB2 and a macrophage marker F4/80 of LSECs isolated from the liver of 129S6 and DBA/2J mice. Nuclei were visualized by DAPI staining. Bar = 20 μm. **e** Quantitative RT-PCR of *Stab2* in the isolated LSEC and macrophage fractions from the livers of 129S6 and DBA/2J. *p < 0.05 vs. 129S6 in LSEC (*n* = 3). Macrophage expression was tended to be higher in DBA, but statistics were not performed because sample size in DBA2J was *n* = 2 while *n* = 3 in 129S6

Despite the high levels of ectopic transcripts, DBA/2J mice show more than ten times higher plasma HA compared to C57BL/6 and 129S6 mice (Kayashima et al. 2015). This phenotype is similar to that of *Stab2*-null mice, where the circulating levels of HA is more than ten times higher than the wild-type controls (Hirose et al. 2012; Schledzewski et al. 2011). Western blot analysis indicates STAB2 protein amounts in the DBA/2 livers are less than 50% of the amount in the 129 livers, despite that they have about equal amounts of *Stab2* transcripts (Fig. 7a). Immunohistochemical analyses detected STAB2-positive cells lining the liver sinusoids of 129S6 mice, whereas the signal intensity was much reduced in DBA/2J mice compared to 129S6 livers (Fig. 7b). For these experiments, we used polyclonal antibodies raised against a peptide corresponding to the cytoplasmic domain of mouse STAB2 (see “Materials and methods”). Since there is no amino acid change in the cytoplasmic domain, it is unlikely that recognition of STAB2^DBA^ protein by the antibodies is affected. Furthermore, although the amount of *Stab2* transcripts in macrophages was reasonable, protein products were undetectable by Western blot analysis, immunohistochemistry, and flow cytometry (not shown). These results suggest that the translation of the LTR-driven *Stab2*^*DBA*^ transcripts must be less efficient than that of *Stab2*^*129*^ or *Stab2*^*B6*^. In addition to the natural initiation codon, the IAP-driven transcript contains one ATG within the IAP sequence and another ATG within the sequence shared with a normal transcript (Fig. 3c). Neither of these upstream ATG codons are in frame with the natural initiation codon, nor have strong KOZAK sequences (Kozak 1987), and the normal ATG site is predicted to be a true translation initiation site by TIS Miner program (https://dnafsminer.bic.nus.edu.sg) (Liu and Wong 2003). Nevertheless, these additional ATG codons could also interfere with correct translation from the transcripts initiated from the 5′LTR, and/or the secondary structure formed by the lengthened 5′ untranslated region may be interfering with the assembly of translational machinery (Leppek et al. 2018).

### Reduced expression of *Stab2*^*DBA*^ in the LSECs

Because the main cell type expressing *Stab2* is liver sinusoidal endothelial cells (LSECs), we isolated these cells by collagenase perfusion and density gradient centrifugation (Meyer et al. 2016). First, the non-parenchymal cell (NPC) fraction which includes LSECs was separated from hepatocytes (HCs), and non-adherent cells were removed by selective adherence. When compared to HCs, the NPCs deprived of non-adherent cells showed higher expression of an endothelial cell maker *Pecam1* (*platelet/endothelial cell adhesion molecule 1*, or *Cd31*), but lower levels of a hepatocyte marker *Alb* (*Albumin*). In contrast, the HCs expressed low *Pecam1* and high *Alb*, confirming the separation of NPCs and HCs (Fig. 7c). Although the expression of *Stab1* mRNA in the NPCs was not significantly different between C57BL/6J, 129S6 and DBA/2J, *Stab2* mRNA was reduced in the NPCs of DBA/2J to less than 50% of those in C57BL/6J and 129S6 (*p* < 0.001). No ectopic expression of *Stab2* was observed in the isolated HCs (Fig. 7c).

We further purified LSECs by eliminating Kupffer cells, a type of tissue-resident macrophage, and other contaminating cells with CD11b^+^ magnetic-activated cell sorting (MACS) followed by selective adherence, according to the protocol by Meyer et al. (2016) (Fig. S3). Before purification, the NPC fraction consists of approximately 50% LSECs, 20% Kupffer cells and 30% unidentified non-adherent cells (Fig. S3, panel (A)). Consistent with the report by Meyer et al., the purification technique allowed final purity of LSECs of more than 93% (Fig. S3, panel (B)). The LSECs isolated from 129S6 mice were positive for immunofluorescence staining of STAB2 but negative for the Kupffer cell marker F4/80 (Fig. 7d). STAB2 staining was also positive in the LSECs of DBA/2J, but the intensity was weaker compared to 129S6 (Fig. 7d). Furthermore, quantitative RT-PCR of the LSECs showed that the expression of *Stab2* mRNA was reduced in DBA/2J to about 25% of the mRNA in 129S6 (*p* < 0.05) (Fig. 7e). In contrast, *Stab2* mRNA levels in the CD11b^+^ fraction were higher in DBA/2J than in 129S6. This is consistent with our previous observation that the mRNA level of *Stab2* in peritoneal macrophages was higher in DBA/2J than in C57BL/6 and 129S6 (Kayashima et al. 2015). Together, these results support our conclusion that normal transcription of *Stab2*^*DBA*^ is reduced in the LSECs, leading to the lower levels of STAB2^DBA^ protein in the LSECs and accumulation of plasma HA in DBA/2J mice.

## Discussion

As the power of the high-throughput sequencing technique evolves, information on genomic variation between species, strains and individuals is rapidly accumulating. However, the biological effects of each variation and the interactions among those variants, which can cause differences in physiological and pathological traits, have not been fully understood. In the current paper, we examined the effects of genetic variations of the *Stab2* gene that are uniquely different in the DBA/2J strain of mice compared with other strains and showed three major findings associated with the *Stab2* gene regulation. Firstly, insertion of an IAP in the promoter region is necessary for the ectopic *Stab2*^*DBA*^expression that starts from the 5′LTR in a reverse orientation. Secondly, this *Stab2*^*DBA*^ transcription is under the control of a second locus at Chr13:59.7–73.0 Mb. The DBA allele at this locus is permissive for the transcription when homozygous, while the C57BL/6J allele dominantly suppresses this expression. Thirdly, the IAP element interferes with the normal gene transcription, causing reduced STAB2 protein levels in LSECs and elevated plasma HA in DBA/2J mice.

Insertions of TEs have taken place frequently during the evolution of mammals, and TEs comprise approximately 40–45% of the mammalian genome (Waterston et al. 2002). Most of the TEs in the mammalian genome are likely suppressed by epigenetic mechanisms, while some TEs escape the suppression and can affect expression of nearby genes and sometimes those several Mb away, in both positive and negative fashion, causing mutant phenotypes (Gagnier et al. 2019). Those differentially fixed in the individual mouse strains could cause strain-specific traits, while insertions in an individual mouse within a strain are often recognized as developmental mutations. One well-known example is the *Agouti viable yellow* (*A*^*vy*^) allele which arose in C3H/HeJ mice and carries an IAP sequence at ~ 100 kb upstream of the *Agouti* gene (Dickies 1962). The wild-type *Agouti* gene products induce the synthesis of yellow pigment by melanocytes during the limited time of the hair growth cycle, producing a yellow band on a black hair (agouti coat color). The antisense oriented 5′ IAP LTR of the *A*^*vy*^ allele drives ectopic expression of the *Agouti* gene throughout the entire hair cycle, which causes a yellow hair (Perry et al. 1994). Because the activity of the IAP is epigenetically regulated by DNA methylation, the coat color ranges from pseudoagouti (IAP is inactive in all cells) to yellow (IAP is active in all cells), and the intermediate mottled pattern of psedoagouti and yellow (mosaic of active and inactive cells), depending on the status of IAP activation (Morgan et al. 1999). Similarly, insertion of IAP causes variable phenotypes in *Axin*^*fu*^, *mCabp*^*IAP*^, *c*^*m*^ and *c*^*m10R*^ (Druker et al. 2004; Porter et al. 1991; Rakyan et al. 2003; Wu et al. 1997).

In this study, we characterized an IAP sequence which is inserted in the promoter region of *Stab2* and upregulates transcription of *Stab2* in DBA/2J mice in the tissues where *Stab2* expression is undetectable in 129S6 or C57BL/6J mice. STAB2 protein from the ectopic mRNA was undetectable, consistent with a previous report that although *Stab2* mRNA was expressed, protein of STAB2 was not observed in P388D1 cells, a monocyte/macrophage cell line derived from DBA/2 mice (Lee et al. 2011). In contrast, total transcription of *Stab2* in LSECs was reduced in DBA/2J, where *Stab2* is normally highly expressed in C57BL/6J and 129S6. Although there is no information available at present on how normal transcription of *Stab2* is regulated in LSECs, it is possible that the IAP sequence disturbs the transcriptional apparatus, or blocks the interaction between the 5′ region of *Stab2* and the promoter/enhancer complex (Whitelaw and Martin 2001). Furthermore, the 5′LTR of the *Stab2*-IAP is rich in CpG dinucleotides, and we showed that the IAP-driven expression of *Stab2* is epigenetically regulated. Currently, there is no cell-culture system available to examine LSEC-specific gene expression, hindering the direct characterization of the regulation of the *Stab2* expression in these cells. Gene expression in primary cells will drift upon placing them in culture, and primary cells are unlikely to be transfected easily. These difficulties need to be overcome to investigate the normal mechanism of *Stab2* gene expression control and the effects of the *Stab2*-IAP in more detail.

Four previous studies have reported the genetic modifiers located on Chr13, which regulate ectopic expression initiated from the LTR of ERV. One is the modifier of dactylaplasia, *Mdac*, which is mapped between 56 to 65 Mb (Kano et al. 2007). Dactylaplasia is caused by an insertion of a MusD in the *Dac* locus on Chr19 and the phenotype is permissible in 129/J, A/J and Balbc/J background which carry *mdac,* but dominantly suppressed by *Mdac* of B6, C3H/J and CBA/J. The second gene, *Clf2*, modifies cleft-palate in A/WySn caused by an IAP1Δ1sequence-driven antisense transcription of *Wnt9b* (*Clf1* on Chr11) (Juriloff et al. 2014). *Clf2* is mapped between 64.95 Mb to 67.9 Mb by an A/WySn x B6 cross, and at least one copy of A-allele is required for cleft-palate to occur, although homozygosity is necessary for full frequency of cleft-palate typical of the A/WySn strain. The third is the sex-limited expression of a complement C4 related gene, *C4a* (*Slp1*), in which an IAP belonging to a class of LTRIS4 is inserted. Krebs et al. reported that two Krüppel-associated box-domain zinc finger protein (KRAB-Zfp) coding genes, regulator of sex limitation 1 and 2 (*Rsl1 and Rsl2*), on Chr13: 67 Mb profoundly affect the strain dependent expression of *Slp1*, which is absent in 129 strains compared to C57BL/6J (Krebs et al. 2003). Finally, Tregger et al. recently reported that *Sgp3* locus in the NZB strain and *Gv1* locus in the 129 strain for the susceptibility to systemic lupus erythematosus phenotypes are linked to 2410141K09Rik and Gm10324 at Chr13: 66 Mb, both of which encode for KRAB-ZFPs (Treger et al. 2019). Unlike very closely related C57BL/6J, C57BL/6N is a strain of mice that uniquely possess a homozygous deletion of 2 Mb, including these two genes and probably other uncharacterized ZFP genes. This deletion renders them unable to repress overproduction of non-ecotropic ERV envelop glycoprotein gp70. This phenotype was not complemented by either NZB or 129 genome.

The region of Chr13 contains a large number of genes for KRAB-ZFP sequences, that play a pivotal role in silencing the expression of ERVs. Their C-terminal zinc fingers recognize and bind to ERV specific DNA sequences while their N-terminal domain interacts with KRAB associated protein 1 (KAP1) to recruit chromatin modifying enzymes that lead to inhibition of ERV-originated transcription. Evolutionally, expansion and diversification of KRAB-ZFP genes coincide with the expansion and insertions of new provirus sequences into the genome, and this relationship has been proposed to be a part of adaptive repressor mechanisms to minimize the potential damage caused by insertional mutations (Ecco et al. 2017). On one hand, a rapid amplification and diversification of genomic sequences can be achieved most effectively through homologous recombination between the repetitive sequences in a gene cluster (Maeda and Smithies 1986). KRAB-ZFP is one of the largest transcription factor gene families in mammals; approximately 500 genes are clustered in 18 locations on multiple chromosomes (Kauzlaric et al. 2017) and are well situated for the prompt silencing of new TE insertions. On the other hand, high copy numbers of homologous genes in a cluster are vulnerable for deletions and gene conversions via homologous recombination during normal replication. Consequently, loss or altered KRAB-ZFP function frequently results in increased variability in their interactions with endogenous TEs and silencer functions. Complex and strain-specific gene rearrangements of the KRAB-ZFP genes neighboring *Rsl1* and *Rsl2* on Chr 13 have been reported (Krebs et al. 2005). Our result strongly indicates that DBA-specific alterations on Chr13 cancel the silencer function on the *Stab2*-IAP-driven transcription. Whether or not any of these modifier loci are common remains to be answered.

We previously suggested that the *Stab2* gene underlies *Arch atherosclerosis 5* (*Aath5*), a quantitative trait locus (QTL) on Chr10 responsible for plaque development in the aortic arch (Kayashima et al. 2015). In the F2 populations of DBA/2J-*Apoe*^−/−^ X 129S6-*Apoe*^−/−^ and of DBA/2J-*Apoe*^−/−^ X C57BL/6-*Apoe*^−/−^, the DBA/2J allele of *Aath5* is protective against plaque development, while the allele shared by 129S6 and C57BL/6J is susceptible (Kayashima et al. 2015; Makhanova et al. 2017). Although the roles of *Stab2* in atherosclerosis have yet to be validated, our results suggest that the DBA-specific genomic alterations restrict *Stab2* functions and cause the accumulation of HA, which supports the idea that *Stab2*^*DBA*^ potentially contributes to protection against early atherosclerosis, since HA infusion in animals is atheroprotective by reducing infiltration of immune cells into plaques (Beldman et al. 2017). On the other hand, low molecular weight-HA has been shown to promote inflammation through binding to alternative receptors including CD44 and could accelerate atherosclerosis (Bot et al. 2010). The effect of *Stab2* inhibition on atherosclerosis is currently under investigation.

In summary, we found that the IAP element in the promoter region of the *Stab2*^*DBA*^ allele alters the expression pattern of *Stab2* in DBA/2J mice. Our experiments showed that the transcription of *Stab2*^*DBA*^ allele is initiated within the 5′LTR, while the insert disturbs the normal transcription of *Stab2*^*DBA*^ in LSECs. The *Stab2*-IAP would provide a new platform to examine the regulation of the IAP and nearby genes. This is a product of random and accidental evolution of mammalian genome, and it is extremely unlikely that human STAB2 has experienced a similar event. There is no report to date on mutations in the human *STAB2* gene that inhibit its function and affect plasma HA. Nevertheless, the human genome is equally rich in transposable elements that influence the expression of nearby genes (Rebollo et al. 2012). Interactions of multiple loci that regulate gene function via affecting TEs such as we have demonstrated here are likely to contribute to the physiological variations in humans and influence the genetic risk of individuals for common complex diseases.

## Electronic supplementary material

Below is the link to the electronic supplementary material.

Supplementary file1 (DOCX 47 kb)

Supplementary file2 (PDF 218 kb)
