# Supplementary material for: Ectopic expression of the Stabilin2 gene triggered by an intracisternal A particle (IAP) element in DBA/2J strain of mice
Source: Mamm Genome. 2020 Jan 7;31(1):2–16. doi: 10.1007/s00335-019-09824-1 (PMC7060167; doi:10.1007/s00335-019-09824-1)
Supplement: Supplementary file 1 — Supplementary file1 (DOCX 47 kb) [file 335_2019_9824_MOESM1_ESM.docx]

**Supporting Information**

**Table S1. List of primers and probes**

| **Method** | **Name** | **Sequences (5’→3’)** |
| --- | --- | --- |
| Promoter region and probe | Promoter 1  Promoter 2 | GACAGATGGATTGCAATATG  CCTCAGGGTGACCTCACGAT |
|  |  |  |
| Cloning of *Stab2*-IAP | 5’a | CATAGCTATAGGAGAGTGACC |
|  | 5’b | GCTGAGTGACAGGATTTCCT |
|  | 3’a | CACTCCTACAGATTCTCCCT |
|  | 3’b | GATCACCTGATCCTTCAAGC |
|  |  |  |
| 5’RACE | Anchor primer fw | GGCCACGCGTCGACTAGT ACGGGIIGGGIIG GGIIG |
|  | Gene specific 1 rv | CACTCCGTCTTGATGGTTAGA |
|  | Anchor primer 2 fw | GGGCACGCGTCGACTAGTAC |
|  | Gene specific 2 rv | TTTCTCGAGGGTGACCTC CGAT |
|  |  |  |
| qPCR | *Pecam1* fw | ACCCCCAGAACATGGATGTA |
|  | *Pecam1* rv | GCTCTCGTTCCCAGAGCTT |
|  | *Pecam1*probe | ACAGAAGTGGAAGTGTCCTCCCTTGA |
|  |  |  |
|  | *Alb* fw | GGCACCAAGTGTTGTACACT |
|  | *Alb* rv | AGCAGACACACACGGTTCAG |
|  | *Alb* probe | CCTTGTGTGGAGGACTATCTGTCTGC |
|  |  |  |
|  | *Stab1* fw | TTCAGCCGCCTATTGACGGT |
|  | *Stab1* rv | TGGGCCACAGCATCGTGAA |
|  | *Stab1* probe | TGGCCTCCTGCCCATGCTTCAGGA |
|  |  |  |
|  | *Stab2* fw | CAGCAAGTTGATACAGGACTC |
|  | *Stab2* rv | TAGGCCAGAAGAGAGTGACT |
|  | *Stab2* probe | CTTGCTGAAAGTCATCACTGACCCCA |
|  |  |  |
|  | *Actb* fw | CTGCCTGACGGCCAAGTC |
|  | *Actb* rv | CAAGAAGGAAGGCTGGAAAAGA |
|  | *Actb* probe | CACTATTGGCAACGAGCGGTTCCG |
|  |  |  |
| Bisulfite sequencing | PCR left  PCR right | GTTTTGGTTTTGGAATGAGGGAT  CCAAACTAAAAAACCACAAAAACTC |
| *Stab2* genotype | rs263539103-U  rs263539103-V | CTGAGAAACAGGTGGCATGT  TATGCCTGCCTGACGGATTA  (170 bp for DBA, 190 bp for B6 and 129) |
| Ch13:64 Mb | rs221034513-C1 | CCTTGATTGCCACTCAGTGT |
|  | rs221034513-C2 | TCGCTCGTATTGGGAAAGGT  (192 bp for DBA, 180 bp for B6 and 129) |
| CGR13:67 Mb | rs235548481-P1  rs235548481-P2 | GCATCTCGCCTTAAACCCTA  GGCTGGAAAGAATCAAGACC  (185 bp for DBA, 170 bp for B6 and 129) |
|  |  |  |

**Supplementary Figures**

**Fig S1. Mapping of the IAP insertion site of the *Stab2^DBA^* allele with genomic Southern blots.** (A) A 500 bp sequence of the 700 bp DNA probe corresponds to 5’ to the insertion site (Black bars) and a 200 bp sequence corresponds to 3’ to the insertion site (in blue). (B) Positions of the hybridizing restriction enzyme fragments of the 129S6 genome are shown above the map derived from the C57BL6 sequence confirm the sites are identical in 129S6 and C57BL/6J. Only the relevant sites are shown. (C) In DBA/2J genome, an IAP element shown in white line is inserted between the Bgl2 sites. Hybridizing fragments are aligned below the restriction map derived from C57BL/6J sequence and the IAP sequence within the *Cdk5rap1* gene of C57BL/6J. The lengths of all fragments match with the map except for the 5’ Bgl2 fragment. This is because a nucleotide difference generates a Bgl2 site in the 5’ region of DBA/2J genome (marked with **) but not in either 129S6 or C57BL/6J.

**Fig S2. Cloning of the 3’ and 5’ ends of *Stab2*-IAP.** (A) Cloning strategy. To clone the 3’ end of the insertion element, genomic DNA from DBA/2J mice was digested with EcoR1 enzyme, followed by self-ligation at a low concentration (5 ng/μl). The self-ligated circular DNAs were PCR amplified using a forward primer corresponding to the sequence near the EcoR1 site in the intron 1 of *Stab2* (5’a, Table S1) and a reverse primer corresponding to the sequence in the promoter region of *Stab2* (5’b, Table S1). The PCR products were purified and sequenced. The same strategy was used to clone the 5’ end of the insertion, except that Pci1 was used instead of EcoR1, and the PCR fragment was generated using primers (3’a and 3’b, Table S1) for sequencing. (B) Sequences alignment of the 5’LTR and 3’LTR of *Stab2*-IAP. IAP sequence is shown in capitals and sequence upstream to the *Stab2* gene in lower case. The lines indicate identical residues. There are two nucleotide substitutions (A to T and G to T) between the 5’LTR and 3’LTR (red boxes). The Bgl2 sites (agatct) flanking the element are underlined. The CAT box, TATA box and Poly (A) addition site are indicated by blue letters.

**Fig S3. Purification of liver sinusoidal endothelial cells (LSECs) from mouse liver.**

Liver cells were digested with type IV collagenase in situ and most of hepatocytes were separated by centrifugation. Non-parenchymal cells were further purified by density gradient centrifugation to obtain the LSEC population according to the protocol by Meyer et al. (33). Right panels show flowcytometry analyses of cell fractions from the 129S6 liver (A) before and (B) after the CD11b+ magnetic-activated cell sorting (MACS) and selective adherence. LSECs are defined as a STAB2-positive and F4/80-negative population. P, pellet; S, supernatant.
