# Supplementary figures and images for: Ectopic expression of the Stabilin2 gene triggered by an intracisternal A particle (IAP) element in DBA/2J strain of mice
Source: Mamm Genome. 2020 Jan 7;31(1):2–16. doi: 10.1007/s00335-019-09824-1 (PMC7060167; doi:10.1007/s00335-019-09824-1)

Figure S1

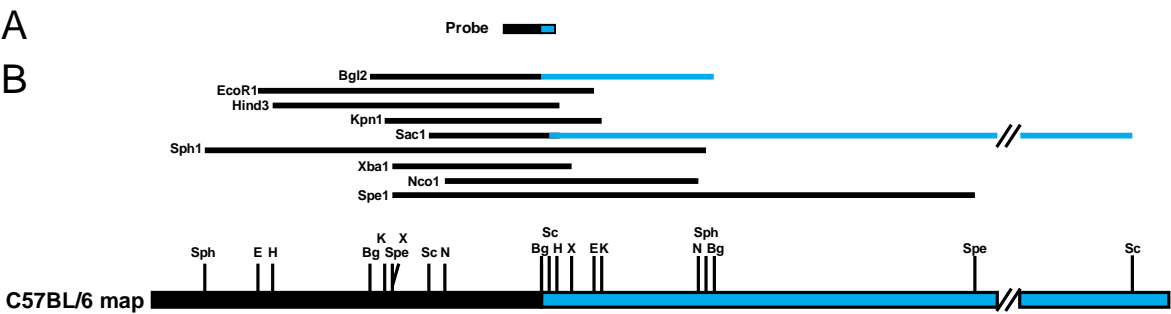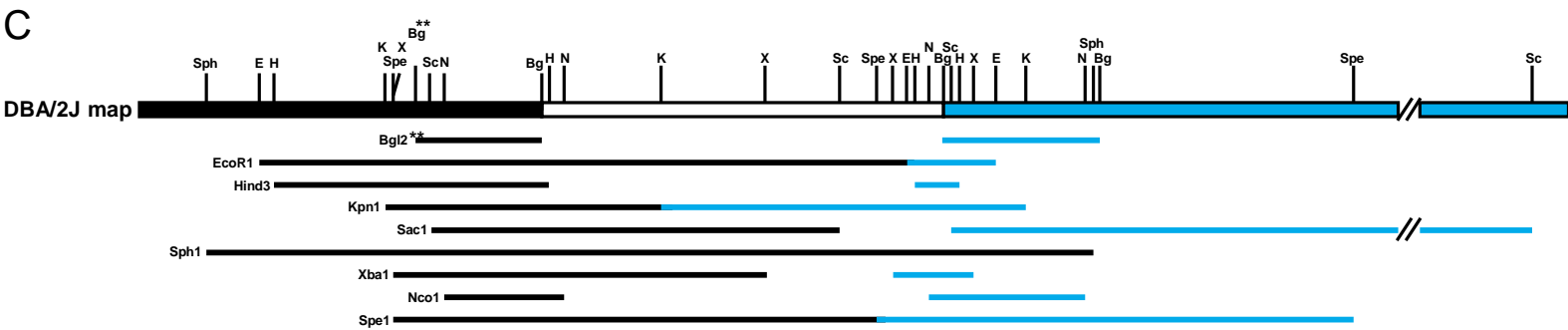

Figure S2

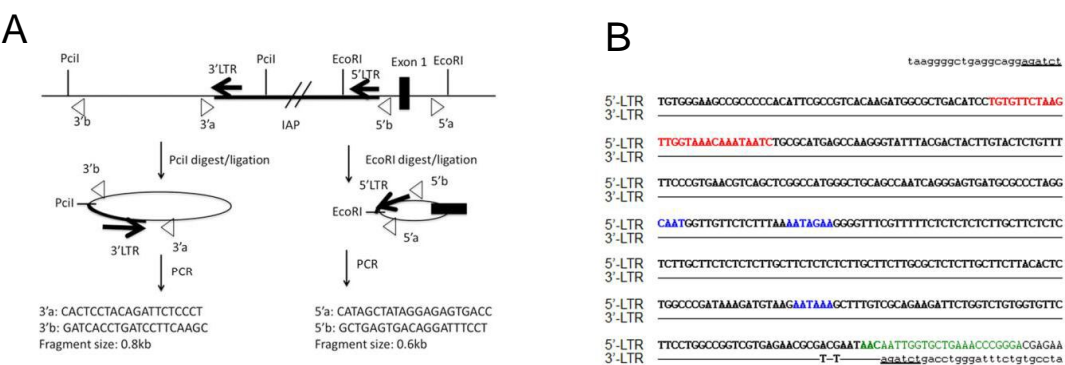

Figure S3

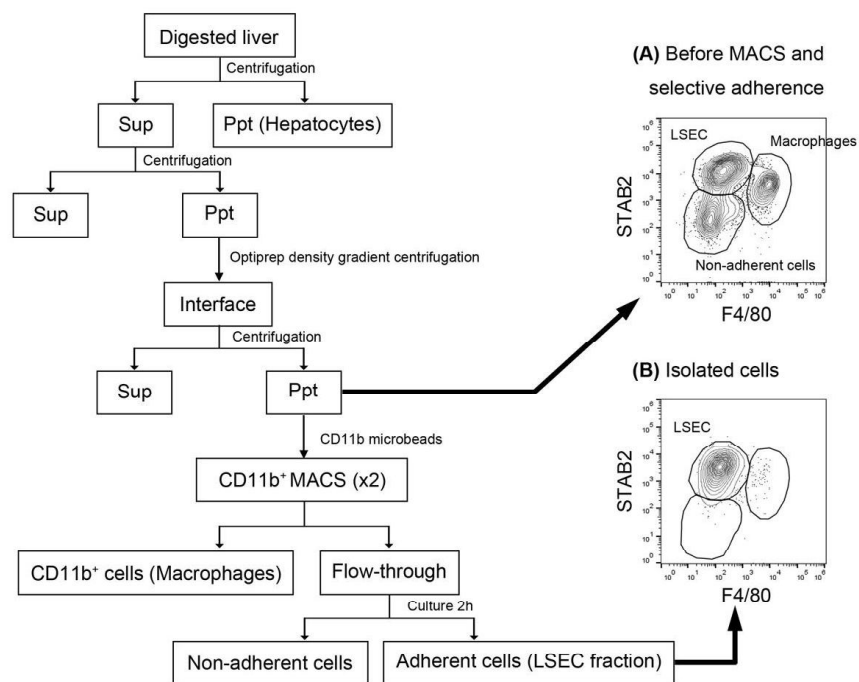

Supplement: Supplementary file 2 — Supplementary file2 (PDF 218 kb) [file 335_2019_9824_MOESM2_ESM.pdf]
